# Corneal decompensation due to spontaneous absorption of lens and anterior dislocation of lens capsule: A case report

**DOI:** 10.1097/MD.0000000000018417

**Published:** 2019-12-16

**Authors:** Shuangqing Wu, Xiaoyu Yu, Qi Dai, Yana Fu, Xiaolei Lin

**Affiliations:** a Department of Ophthalmology, Zhejiang Provincial Integrated Chinese and Western Medicine Hospital, Hangzhou; b Eye Hospital, Wenzhou Medical University, Wenzhou, Zhejiang Province, China.

**Keywords:** corneal decompensation, lens absorption, lens dislocation

## Abstract

**Rationale:**

Spontaneous absorption of lenses or cataracts is rare. However, lens capsule attachment to the endothelium combined with corneal decompensation can still occur.

**Patient concerns:**

An 81-year-old male presented with left eye pain and bulbar conjunctival injection for 6 months. Diffuse corneal edema and inferior bullous lesions were observed by slit-lamp microscopy. Following examination with swept-source optical coherence tomography, we could clearly identify a membrane structure adherent to the corneal endothelium, as well as a lens not in situ. In vivo confocal microscopy found decreased corneal endothelial density of 745 ± 46 cells per mm^2^ in the left eye.

**Diagnosis:**

Lens dislocation and spontaneous absorption, combined with corneal decompensation were diagnosed.

**Interventions:**

Surgical removal of the membrane structure combined with anterior vitrectomy was performed.

**Outcomes:**

The patient's symptoms were partly relieved. However, the corneal endothelial decompensation could not be entirely reversed. In vivo confocal microscopy verified that corneal endothelium was in situ and the density was not significantly changed in the left eye.

**Lessons:**

This case study reports a rare dislocation and spontaneous absorption of lens without any trauma or subsequent surgery. Moreover, it demonstrates corneal endothelial decompensation due to the lens capsule adhering to the corneal endothelium. Timely intervention is required to remove the dislocated lens and prevent complications.

## 1 Introduction

Corneal decompensation is usually induced by surgery, trauma, dystrophy, or infection.^[1]^ In this study, we describe a patient who developed corneal decompensation due to a membrane adhering to the endothelium. He was diagnosed with cataract 40 years ago. In addition, the patient had a history of sudden improvement of vision 30 years ago. Hence, disappeared lens, lens dislocation, and spontaneous absorption were considered for this case.

## 2 Case presentation

An 81-year-old male presented at Wenzhou Medical University Eye Hospital with a complaint of ocular pain and bulbar hyperemia in the left eye persistent in the past 6 months. He had a history of unexplained cataract in his left eye for > 40 years, and had once experienced a sudden bright light 30 years ago without any other signs of discomfort and did not receive any treatment. The patient had no history of trauma, surgery, and ocular diseases. Moreover, there was no family history of eye disease, and the patient did not suffer from any systemic diseases besides hypertension.

**Figure 1:**
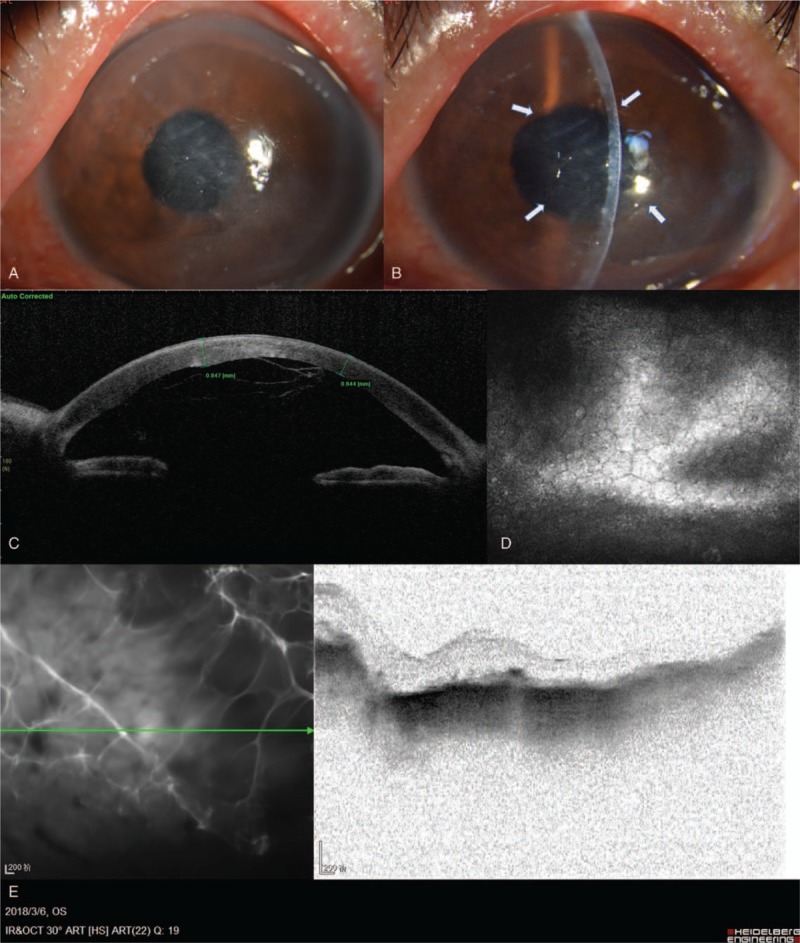
Preoperative manifestations of the left eye. (A) Slit lamp biomicroscope showed widespread corneal edema and inferior bullous lesions. (B) Arrows indicate a translucent membrane with a rolled-up margin attached to the posterior surface of the cornea. (C) SS-OCT image illustrates that the membrane in the anterior chamber appears to be a lens capsule which clung to the posterior corneal surface. (D) Confocal microscopy of the corneal endothelium shows altered size and morphology. (E) OCT exhibits choroid atrophy and subretinal hyper-reflection with medial opacity.

At his initial examination, visual acuity was 20/40 in the right eye and hands moving before eye in the left eye, with no improvement of visual acuity by correction. Intraocular pressures were 12.6 and 6.8 mmHg in the right and left eyes, respectively. Slit-lamp microscopy revealed diffuse corneal edema and a localized area of bullous lesions in the inferior quadrant of the left eye (Fig. 1A). A translucent membrane with a rolled-up margin could be seen indistinctly adhered to the endothelium in the pupillary zone (Fig. 1B). The anterior chamber was deep with herniated vitreous, whereas the crystalline lens was absent. Apart from moderate cataract, the right eye was essentially within normal limits.

Ultrasound B-scan (Aviso, Quantel Medical, France) of the left eye revealed a normal posterior segment without any significant findings, such as a dislocated lens. Axial lengths were found to be 23.90 and 23.21 mm in the right and left eyes, respectively, using optical biometry (IOL-Master 500, Carl Zeiss, Germany). Swept-source optical coherence tomography (SS-OCT; SS-1000, Tomey Corporation, Japan) showed a multilayer membrane in the anterior chamber that clung to the posterior corneal surface (Fig. 1C). In vivo corneal confocal microscopy (HRT 3, Heidelberg Engineering GmbH, Germany) displayed alteration in the size and morphology of the corneal endothelial cells, with cell counts of 745 ± 46 cells per mm^2^ in the left eye (Fig. 1D). Optical coherence tomography (OCT, Heideiberg Spectralis OCT, Heidelberg Engineering GmbH, Germany) showed choroid atrophy and subretinal hyperreflection with medial opacity (Fig. 1E).

**Figure 2:**
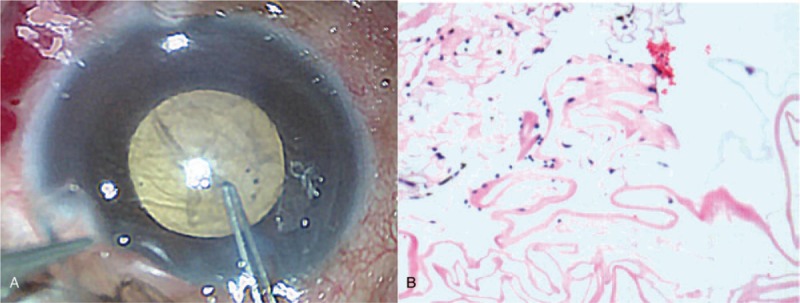
Intraoperative image of the left eye and histopathology of the membrane structure. (A) Intraoperatively, the membrane structure was extracted. (B) Histopathology shows the membrane structure consisting fibrous tissue with a few pigment cells.

**Figure 3:**
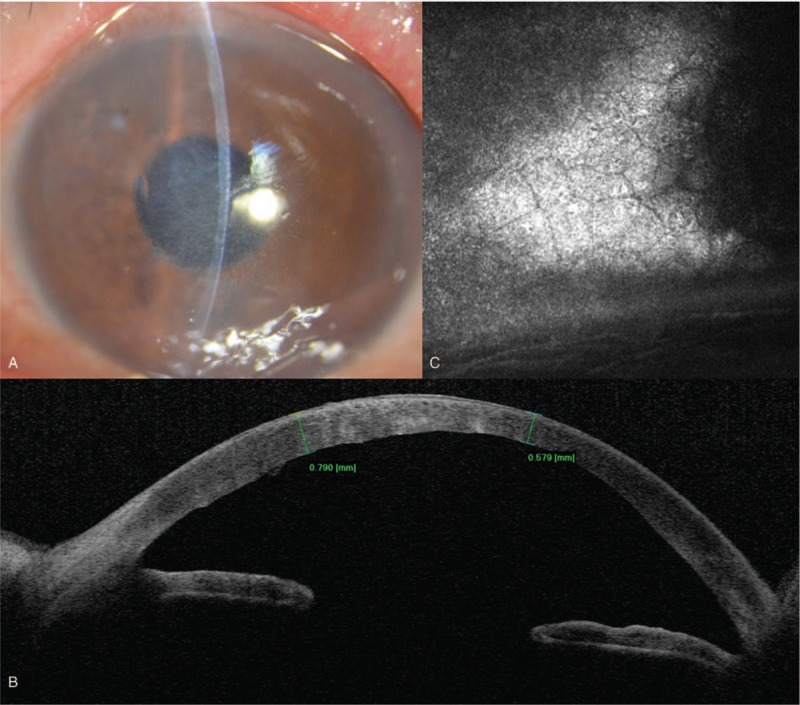
Postoperative manifestations of the left eye at 3-month. (A) Partly relieved corneal edema under slit-lamp biomicroscope. (B) SS-OCT demonstrates a partly relieved corneal edema. (C) Confocal microscopy of the corneal endothelium showing no changes.

The patient underwent membrane extraction and anterior vitrectomy on the following day. The anterior chamber was penetrated by a corneoscleral incision at 11 o’clock. During the perioperative process, we identified that the membrane was tightly adhered to the endothelium (Fig. 2A). Histopathology demonstrated that the membrane structure consisted of fibrous tissue with a few pigment cells (Fig. 2B). No evidence of dislocated lens, nucleus fragments, or any cortical materials was found intraoperatively. Although ocular pain was resolved, and conjunctiva hyperemia and corneal edema were alleviated, the visual acuity and corneal endothelium decompensation could not be reversed 3 months after surgery (Fig. 3). In vivo confocal microscopy revealed that the corneal endothelium was in situ and that the left eye density was not significantly changed (Fig. 3).

## 3 Discussion and conclusions

In the present case study, the patient was diagnosed with left eye cataract > 40 years ago. Lens dislocation might have occurred when he experienced a sudden bright light 30 years ago without any other signs of discomfort. Typically, lens dislocation due to zonular weakness can be induced by trauma, intraocular surgeries, eye diseases (such as pseudoexfoliation syndrome, high myopia, retinitis pigmentosa, uveitis, endophthalmitis, and intraocular tumor), or hereditary disorders (such as microspherophakia, Marfan syndrome, and Weill-Marchesani syndrome).^[2–4]^ Our patient did not have any history of trauma or surgery, and no pseudoexfoliation was observed in either eye. In addition, there was no pertinent evidence regarding the aforementioned etiologies for this patient.

It has been reported that spontaneous capsule rupture and lens dislocation can occur in hypermature cataract.^[5]^ Since the patient recalled experiencing a sudden bright light over 10 years following his cataract diagnosis, we speculated that the lens was totally dislocated because of the hypermature cataract that day, accompanied with lens protein liquefaction and its absorption over a long period without any discomfort.

Spontaneous lens absorption is rare, but it can occur in cases of hypermature cataract,^[5]^ traumatic capsular rupture,^[6]^ rubella-related congenital cataracts ^[7]^ and leptospiral uveitis.^[8]^ This process typically involves capsular rupture, crystalline protein absorption, and residual empty capsular bag formation. The exact mechanism of lens absorption remains unclear; hence, clinical manifestations are bound to vary according to the underlying cause. In the present study, only a translucent membrane was left and pushed by the herniated vitreous body to the rear surface of the cornea. Histopathology confirmed that it was fibrous tissue with a few pigment cells. The tissue shape revealed by SS-OCT in combination with the integrated endothelium following operation allowed us to consider that it was the empty capsular bag. Two case reports have described spontaneous lens absorption and the empty capsular bag dislocated into the anterior chamber in unilateral^[9]^ or bilateral eyes.^[10]^

Idiopathic lens luxation into the anterior chamber accompanied with corneal endothelial cell loss within several weeks has been reported previously.^[11]^ Spontaneous lens absorption and dislocation of capsular bag into the anterior chamber seemed to have no impact on corneal endothelium in previous cases.^[9,10]^ The capsule membrane contiguity to the corneal endothelium was much loose in Kim's case,^[9]^ and the lens capsular might dislocate from the posterior chamber to the anterior chamber following spontaneous crystalline protein absorption in situ. According to the hypothesis developed by Kim et al,^[9]^ the mechanism of lens capsular dislocation from the posterior chamber to the anterior chamber probably stems from a temporary in pressure caused by eyelid squeezing, which induces a pressure gradient across the pupil. However, in the present case, irreversible corneal endothelium decompensation occurred, which may be associated with lens dislocation prior to lens absorption. Furthermore, because of the patient's age and vitreous liquefaction, lens dislocation was accompanied with herniated vitreous body. The tight contiguity of the capsule membrane to the rear surface of the cornea has the capacity to block the contacting between aqueous humor and endothelium, and thus affect corneal endothelial metabolism. Moreover, our patient was significantly older compared with those in Kim's and Ahmad’ cases,^[9,10]^ inclining to occur corneal endothelium decompensation.

Symptoms of pale optic disc and sheathing of the retinal vessels were found in Ahmad's case.^[10]^ We also detected choroid atrophy and subretinal hyper-reflection with medial opacity by OCT. It is common knowledge that spontaneous crystalline protein absorption tends to induce inflammation, accompanied with uveitis and secondary glaucoma.^[5]^ Although our patient did not complain of any discomfort or symptoms in the past 30 years, occult chronic inflammation may be associated with corneal endothelial damage and retinopathy. In addition, aged-related degeneration of the fundus should also be considered.

We report a case of corneal endothelial decompensation due to dislocation and spontaneous absorption of lens, and the lens capsule adhered to the corneal endothelium 30 years following lens luxation. This rare case illustrates the serious consequences of cataract without early surgical intervention. Therefore, timely and appropriate medical intervention is of utmost importance to prevent these complications.

## Author contributions

**Conceptualization:** Yana Fu.

**Data curation:** Xiaolei Lin.

**Funding acquisition:** Shuangqing Wu.

**Supervision:** Qi Dai.

**Writing – original draft:** Shuangqing Wu, Xiaoyu Yu.

**Writing – review & editing:** Qi Dai.
